# The hundred person wellness project and Google’s baseline study: medical revolution or unnecessary and potentially harmful over-testing?

**DOI:** 10.1186/s12916-014-0239-6

**Published:** 2015-01-09

**Authors:** Eleftherios P Diamandis

**Affiliations:** Department of Pathology and Laboratory Medicine, Mount Sinai Hospital, Toronto, Ontario M5T 3L9 Canada; Department of Clinical Biochemistry, University Health Network, Toronto, Ontario M5G 2C4 Canada; Department of Laboratory Medicine and Pathobiology, University of Toronto, Toronto, Ontario M5S 1A8 Canada; Head of Clinical Biochemistry, Mount Sinai Hospital and University Health Network, 60 Murray St. Box 32, Floor 6, Rm L6-201, Toronto, ON M5T 3L9 Canada

**Keywords:** Cancer screening, Incidental findings, Indolent disease, Over-diagnosis, Over-treatment, Population screening

## Abstract

The Hundred Person Wellness Project is an ambitious pilot undertaking, which aims to intensely monitor 100 individuals over 10 months. Patients with abnormal findings will be treated, in hopes that this early intervention will avoid, or delay, symptomatic disease. Google’s “Baseline Study” is of similar scope and will enroll 10,000 people over 2 to 3 years. I here speculate that these approaches will likely not be effective in preventing disease, but instead, lead to unnecessary and potentially harmful interventions. Examples from the cancer screening experience over the last 30 years are provided, which show that intensive testing may uncover indolent disease or incidental findings which, when treated, may cause more harm than good. Additional examples show that aggressive treatments for cancer and other diseases do not always lead to better patient outcomes. I conclude that the recent advances in omics provide us with unprecedented opportunities for high content clinical testing, but such testing should be used with caution to avoid the harmful consequences of over-diagnosis and over-treatment. Despite the detailed rebuttals by Hood and colleagues in another commentary in *BMC Medicine*, time will show the actual benefits and harms of these ambitious initiatives.

Please see related commentary: http://dx.doi.org/10.1186/s12916-014-0238-7

## Background

A new project has been undertaken by Dr. Leroy Hood, President of the Institute for Systems Biology, Seattle, USA. The Hundred Person Wellness Project (HPWP) [1] is summarized as follows, starting March 2014, and for 9 months, 100 healthy volunteers will be intensely checked through continuous monitoring (sleep patterns, pulse, physical activity) or with a battery of ~100 biochemical tests in blood, saliva, urine, and stool (every 3 months) [2]. Whole genome sequencing and microbiome ecology assessment will also be performed at the beginning of the study. Additional tests are planned to be added at a later phase. Data will be available to coaching staff as well as to the individuals themselves, and the tests may trigger medical treatments or changes in dietary habits before symptoms appear. If successful, this project aims to expand to 100,000 people within the next 4 years with continuous monitoring up to 30 years. Ambitious project, indeed.

This study design will not include controls. Hood believes that each person will also serve as their own control.

In a similar but unrelated effort, undertaken by Google X, the research arm of Google, approximately 10,000 volunteers are expected to be enrolled and monitored with biochemical and other testing over 2 to 3 years to identify early, asymptomatic disease. The study, known as “the Baseline Study”, will be led by Dr Andrew Conrad and it is a collaboration between Google, Stanford University, and Duke University. For more details see the cited link [3].

In an era of evidence-based medicine, we should examine if testing of asymptomatic individuals (which is the same as population screening; despite disagreement by Hood et al. [4]) has already contributed to better clinical outcomes, and for which diseases. We should further examine if the HPWP and the Baseline Study are in accord with established principles of population screening. To substantiate my statements, I will borrow lessons learned over the last 30 years from cancer screening efforts. Apart from cost, we should keep in mind that successful programs should lead to benefits that outweigh harms (Box 1).

## Discussion

### Principles of population screening

One of the most widely established screening programs includes neonatal screening for phenyl ketonuria and congenital hypothyroidism, introduced more than 50 and 25 years ago, respectively. Advances in mass spectrometry allowed for neonatal screening to expand to approximately 50 rare disorders [5]. The most universally accepted criteria for screening are summarized as follows: the condition should be an important problem with known natural history, and have an agreed policy on whom to treat; diagnostic and treatment facilities should be available; there should be a suitable test; and the cost of case-finding should be economically balanced in relation to medical costs as a whole [5]. Nearly none of these criteria are met by the proposed HPWP or the Baseline Study, since no specific disease is targeted, available and effective medical treatments have not been defined, and no cost analysis exists since the benefits and harms are unknown.

One well-recognized difficulty with neonatal screening (and the situation is very similar with many adult diseases; see below) is that screening may uncover not only clinically significant cases which can benefit from early treatment, but also, a surprising number of cases with positive screening results which do not differ from those of clearly affected cases, but could remain asymptomatic [5]. Such positive results, but of uncertain significance, leave the parents wondering what to do and to situations where treatment is given but is not needed (over-treatment), adding anxiety and costs of unnecessary clinical care. It may be the case that such indolent or incidental findings could lead to over-treatment of many participants of the HPWP and the Baseline Study.

### Biochemical profiling versus discrete testing

Continuous flow analysis (CFA), discovered in 1957, had the ability to simultaneously measure hundreds of analytes in biological fluids. At that time, it was thought that CFA could be invaluable in revealing biochemical changes of early disease signs (an idea similar to the HPWP and the Baseline Study). However, it was quickly realized that such analysis would also yield 5% false-positive tests (i.e., results outside the reference intervals in otherwise normal subjects). This is due to the general definition of reference intervals as 2.5 to 97.5% of values in a reference (normal) population. The high cost of investigating seemingly abnormal results catalyzed the elimination of such biochemical profiling from clinical practice, in favor of discrete testing (meaning that assays are performed only if requested specifically by a physician). CFA will not be used in the HPWP or the Baseline Study; however, as with any profiling strategy, there will be a small percentage of healthy individuals that will have abnormal results, which could lead to additional unnecessary investigations and probably harmful interventions (Box 1).

### Whole genome sequencing (WGS)

WGS has achieved a major milestone in 2014; the $1000 genome. While participants in the HPWP will receive whole genome sequencing, it is not mentioned how this information will be used. As we have described elsewhere [6], there are still many debated issues (technological, quality assurance, interpretative, ethical, and, most importantly, efficacy). It seems that WGS in the HPWP will be used to assess disease risk predisposition, so that preventative measures (if any) or therapeutic interventions can stop or slow down disease processes such as Alzheimer’s disease. Disease predisposition is currently assessed by identifying alleles (single nucleotide polymorphisms) associated with lower or higher risk for developing a disease in a lifetime. Direct-to-consumer testing for predicting disease predisposition became popular a few years ago, but the US Food and Drug Administration has imposed restrictions until the efficacy of the test is proven [7]. The analyses of Roberts et al. [8], derived from data of monozygotic twins, have shown that WGS will likely have major limitations in predicting disease predisposition. While WGS has been proven successful in molecularly characterizing inherited diseases, in prenatal screening and in individualization of treatments and pharmacogenomics, such applications are not relevant to the HPWP or the Baseline Study since participants are all asymptomatic.

### The microbiome

The human microbiome is the population of more than 100 trillion microorganisms that live in our gut, mouth, skin, and elsewhere in our bodies. These microbial communities have numerous beneficial functions such as digestion of food, defense, and synthesis of essential nutrients and vitamins [9]. Despite recent advances in our understanding of the microbiome and its relation to human diseases, we do not as yet have any validated ways of altering the microbiome for effective treatments [10]. Until such manipulations are shown to be safe and effective, their use is not warranted.

### Cancer screening

The premise for cancer screening is that if cancer is detected early, when the lesion is small and localized, the chances of removing it completely, or treating it effectively, are higher; thus, screening should lead to better clinical outcomes. One caveat with population screening is that even if the screening method is highly sensitive (i.e., is detecting most cancers) and highly specific (i.e., results are negative in most healthy individuals), if the disease under consideration is rather rare, the positive predictive value of the test will be low. In such cases, screening usually identifies a lot more false-positives than true-positives. Separating true- from false-positives is not trivial and it may necessitate invasive and potentially harmful procedures such as biopsies, laparotomies, or other major surgeries. An additional major complication of screening is that it uncovers forms of the disease which are deemed to be indolent, meaning that these lesions will not pose a threat to a patient’s life and they will grow slowly and likely remain undetected for long periods (over-diagnosis). When detected, such lesions are usually treated, adding to the cost of health care and putting patients in a lot of stress and probably inflicting serious side effects (over-treatment). Thus, as mentioned earlier, participants in the HPWP and the Baseline Study will likely be subjected to unnecessary follow-up testing or receive treatments that are not really required.

### Successes and failures of cancer screening

A notable example of a successful cancer screening program includes cervical cancer [11]. Screening for colorectal cancer has also shown an approximate 30% lower risk of death [12]. However, colonoscopy-guided screening carries a complication rate of about 0.1%, including colon perforation and bleeding.

For breast cancer, for which there is a 30 year screening experience, it was recently estimated that the 30% decrease in the rate of death from breast cancer is attributed more to improved treatments rather than to screening [13]. It has been estimated that less than 10% of early-stage breast cancers diagnosed by screening progress to advanced disease. This has led to the conclusion that over-diagnosis of breast cancer (i.e., tumors detected by screening that would have never led to clinical symptoms) was approximately 1.3 million women in the past 30 years. Based on this data, it has been suggested that breast cancer screening should be abolished [14].

Lung cancer screening with low-dose thoracic computed tomography of heavy smokers, reduces mortality from lung cancer by approximately 20% [15]. However, false-positive results occur in a substantial proportion of the screened population; 95% of all positive results do not lead to diagnosis of lung cancer. The proportion of invasive diagnostic procedures in patients with one or more lung nodules is approximately 1 to 4%. The risk of major complications is 4.5 per 10,000 persons screened and 25% of the surgical procedures in the nation’s lung screening trial were performed on nodules that were later determined to be benign. Thus, the relatively small benefits need to be weighed against the costs, harms of exposure to radiation, and the vast number of individuals who have benign nodules and invasive follow-up procedures.

For ovarian cancer we do not as yet know the effect of screening on mortality [16]. However, preliminary data reveal that the positive predictive value ranges from 3 to 35%. This means that for confirmation of diagnosis, more women without ovarian cancer will undergo invasive surgical procedures (such as laparotomy) than patients with ovarian cancer.

Despite the enthusiastic endorsement of screening for prostate cancer in the 1990s and 2000s, the prospective randomized clinical trials and meta-analyses have shown that the incidence of prostate cancer in the screening group has increased significantly. One of the studies demonstrated that screening improves risk of prostate cancer-specific death but an additional 37 men needed to receive a diagnosis through screening, for every 1 saved prostate cancer death, after 11 years of follow-up [17,18]. The harms associated with screening include false-positive results, over-diagnosis, over-treatment, and complications of biopsy and treatment. Among men who are undergoing prostatic biopsy, 75% of them do not have cancer; side effects include pain, fever, hematuria, hematochezia, and hematospermia. Side effects of radical prostatectomy include incontinence and erectile dysfunction. Other harms include anxiety and depression. The PIVOT trial has shown that among men with localized prostate cancer, randomized to receive radical prostatectomy or active surveillance, mortality was approximately the same after 10 years of follow-up [19].

The relevance of this data to the HPWP and the Baseline Study is obvious. In addition to over-diagnosis and over-treatment, we should also consider that more aggressive treatments for cancer and other diseases do not always lead to improved patient outcomes.

### Future direction and conclusions

While predictions about the future are difficult, I could hazard to say that the HPWP and the Baseline Study will likely be unable to prove or disprove anything concrete. A few concluding remarks are warranted.

First, it appears that some older and seemingly obvious dogmas, suggesting that serious progressive diseases (such as cancer) can be better treated with more radical surgical procedures and intensive adjuvant supplements, such as radiotherapy/chemotherapy, do not seem to always hold true. At least for prostate cancer, active surveillance (observation), is equivalent to highly invasive surgical procedures such as radical prostatectomy, especially for patients with early stage and grade disease, discovered by screening. Therefore, the previously practiced philosophy of early intensive treatments is now changing to a more conservative approach, at least for some diseases.

The evolution of many omics technologies and modern imaging are giving us unprecedented opportunities to monitor hundreds, or even thousands, of proteins in biological fluids as well as delineate whole genomes and exomes. The cost and turnaround times of such testing are rapidly declining. Consequently, these technologies will surely find their niche in the clinic. However, as I have discussed, more testing, especially of asymptomatic individuals, not only does not guarantee benefit, but, in fact, it may be harmful (Box 1).

## Box 1 Possible benefits and harms of population screening

**Benefits**

- Identification of disease predisposition or early diagnosis, leading to prevention or effective therapy.

**Harms**

- If no treatment or prevention available, diagnosis may cause anxiety/depression.
- False-positives leading to more testing; some testing may be invasive or have side effects (biopsies, surgeries, anxiety, depression).
- Incidental findings/indolent disease^1^ (over-diagnosis, over-treatment, and some treatments may be invasive, have serious side effects, and be costly).
- Harms of testing (e.g., radiation, bleeding, colon perforation).
- Cost effectiveness.

^1^Incidental finding is defined as a finding that is unrelated to the primary reason of patient testing. Indolent disease is defined here as a disease detected by screening that would have otherwise not be detected in a patient’s lifetime.

## Abbreviations

CFA: Continuous flow analysis
HPWP: The Hundred Person Wellness Project
EGS: Whole genome sequencing
